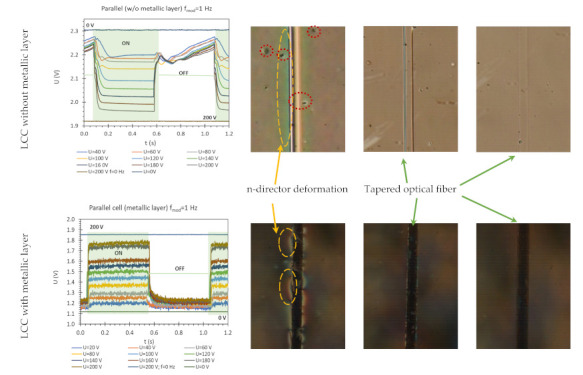# Enhancement of the SPR Effect in an Optical Fiber Device Utilizing a Thin Ag Layer and a 3092A Liquid Crystal Mixture

**DOI:** 10.3390/molecules26247553

**Published:** 2021-12-13

**Authors:** Joanna Korec, Karol A. Stasiewicz, Katarzyna Garbat, Leszek R. Jaroszewicz

**Affiliations:** 1 Institute of Applied Physics, Military University of Technology, 2 Kaliskiego St., 00-908 Warsaw, Poland; karol.stasiewicz@wat.edu.pl (K.A.S.); leszek.jaroszewicz@wat.edu.pl (L.R.J.); 2 Institute of Chemistry, Military University of Technology, 2 Kaliskiego St., 00-908 Warsaw, Poland; katarzyna.garbat@wat.edu.pl

**Keywords:** surface plasmon resonance, silver layer, tapered optical fiber, optical fiber device, liquid crystal device

## Abstract

This paper is a continuation of previous work and shows the enhancement of the surface plasmon resonance effect in a tapered optical fiber device. The study investigated liquid crystal cells containing a tapered optical fiber covered with a silver nanolayer, surrounded by a low refractive index liquid crystal in terms of the properties of light propagation in the taper structure. Silver films with a thickness of *d* = 10 nm were deposited on the tapered waist area. Measurements were performed at room temperature; liquid crystal steering voltage U from 0 to 200 V, with and without any amplitude modulation with a frequency of *f* = 5 Hz, and the wavelength *λ* ranged from 550 to 1200 nm. A significant influence of the initial arrangement of liquid crystals molecules on light propagation was observed. Three types of liquid crystal cells—orthogonal, parallel, and twist—were considered. During the measurements, resonant peaks were obtained—the position of which can also be controlled by the type of liquid crystal cells and the steering voltage. Based on the obtained results, the best parameters, such as highest peak’s width reduction, and the highest SNR value were received for twisted cells. In addition, the present work was compared with the previous work and showed the possibility of improving properties of the manufactured probes, and consequently, the surface plasmon resonance effect. In the presented paper, the novelty is mainly focused on the used materials as well as suitable changes in applied technological parameters. In contrast to gold, silver is characterized by different optic and dielectric properties, e.g., refractive index, extension coefficient, and permittivity, which results in changes in the light propagation and the SPR wavelengths.

## 1. Introduction

Surface plasmon resonance (SPR) sensors are widely used to analyze human fluids such as blood, urine, as well as to detect vitamins, e.g., B3, K1, A [1,2,3,4], proteins [5], also, for chemical analyses [6,7], and environmental screening especially for contamination from biological toxins and pathogens [8,9]. Contrary to typical sensors, the main advantage of optical probes is that they are cheap, neutral for the tested environment, do not need reference electrodes, and are not affected by electromagnetic interference [10].

Fiber optic plasmonic sensors (FOPS) are probes based on the generation of plasmons that use thin metal films and nanostructures [11]. To allow a guided wave to interact with the sensing medium, an optical fiber geometry should be modified [7]. Consequently, several types of probes can be mentioned among the FOPS due to their geometry. The first one is based on modifications placed on the optical fiber face and includes angle-polishing, mirrors, and tips [12,13,14]. The second one is the modification of a side surface including side polishing, etching, bending, tapering, and making hetero-cores [15,16,17,18,19]. In this paper, the tapered optical fiber (TOF) was investigated and only this modification will be taken into consideration.

**Figure 1:**
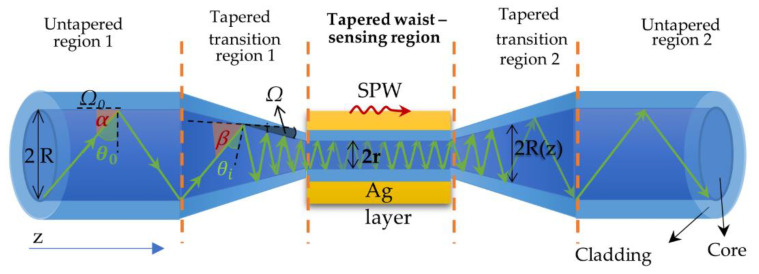
Optical fiber-based SPR probe with a uniform waist area sandwiched between two tapered transition regions, where: *Ω* is the tapered fiber angle; *R* is the diameter of the untapered fiber; r is the diameter of the tapered waist area*; θ*_0_ is the angle of ray incidence; *α* and *β* are the angles with the fiber axis (from \alpha to \beta).

From a sensory point of view, the highest impact on sensitivity and utility of surface plasmon resonance (SPR) sensor types have overlays and a probe geometry [20,21]. Basically, tapers are simple devices, however, their theoretical description and fabrication are very challenging, especially regarding repetitive properties [22]. The manufacturing process is based on the uniform stretching of a fiber over a high-temperature burner [23,24]. As a result of this process, different types of tapers can be obtained: punctual (without a uniform waist area), short, or long (with a short or long uniform waist area) [23,25]. It was proven that even a small elongation and a change in the angle of incidence increased the sensitivity. For sensors applications, the best results are obtained for tapered optical fiber probes which contain a long-tapered waist area between two tapered transition regions enabling coupling and transformation of power from or to a nontapered taper with small insertion losses known as the adiabatic shape [26]. Since tapers and SPR sensors can be manufactured from different types of optical fibers, higher sensitivity is typically attributed to single-mode fibers with a lower refractive index [11,21]. The TOF scheme with a uniformly tapered waist is presented in Figure 1.

During elongation, the change occurs in the geometry (parameters *R*, *Ω*) of the taper transition region (1 and 2), as well as in the tapered waist diameter from 2*R* to 2*r*. All these parts play a significant role during light propagation. The purpose of taper transition region 1 is to reduce the ray incidence angle with the normal to the core-cladding interface from $\theta_{0}$ to $\theta_{1}$ or from *α* to *β* along the tape boundary [27]. If the adiabaticity condition of the taper is met, most of the optical power travels inside the fiber with an angle close to critical. As a result, the penetration depth of the evanescent field increases to almost the maximum value [28,29]. Taper transition region 2 returns the angles of these rays to their initial values.

It is well known that decreasing the core diameter influences the increasing number of reflections per unit of any ray. However, a larger number of reflections increase the peak width what is highly undesirable. On the other hand, a smaller diameter of the tapered waist provides sensitivity enhancement. Thus, changing the profile transition regions and maximizing the ratio compensates for this disadvantage [29,30]. In Figure 1, for visualisation of changes taking place in the fiber light, reflections as rays are presented. A detailed description of light traveling through the graded-index single-mode taper fiber as a Gaussian shape beam is presented in the previous literature [31].

As mentioned earlier, the type of covering material has a significant influence on the probe sensing parameters: sensitivity, detection accuracy, and signal-to-noise ratio (SNR) $SNR=\frac{\delta \lambda_{res}}{\Delta \lambda_{0.5}}$ where: *δλ_res_* is the resonant dip shift resolution; Δ*λ*_0.5_ is the resonant dip width measured at a 0.5 peak height. To obtain the best SPR sensor, all these factors should be as high as possible [32]. In the case of metallic covers, the mentioned parameters depend on the value of dielectric permittivity *ε* of the metal used. Metals have a complex dielectric constant of *ε = ε_r_ + iε_i_*. The real part *ε_r_* is related to the number of reflections and the imaginary part *ε_i_* is responsible for the absorption. The SPR spectrum is very sensitive to the values of both components where the sharpness of the resonant dip is related to the *ε_r_/ε_i_* ratio. When this ratio increases, a dip sharpness also increases [33]. Moreover, the type of cover used determines the spectral regime on which the probe will be operating. Table 1 includes parameters of typical metals used to cover the SPR probes. There are various metals that respond to an SP wave as well as the electromagnetic coupling relationship [34].

**Figure 2:**
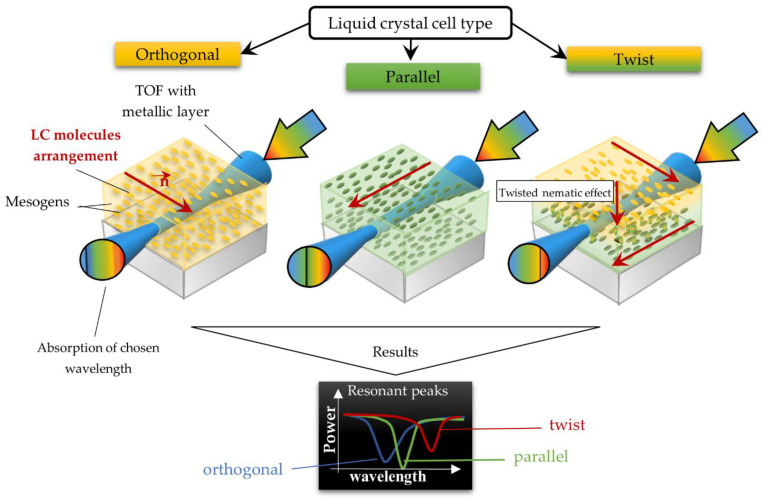
LCCs types with a sputtered metallic layer in the tapered area, in relation to the mesogens arrangement inside the cell. The black block shows samples of resonant peaks obtained for LCCs: orthogonal, parallel, and twist.

Figure 2 presents a schematic diagram of the liquid crystal cell (LCC) with a different initial molecule arrangement: orthogonal, parallel, and twist corresponding to the taper/fiber longitudinal axis. Other technological aspects of cell preparation can be found in a previous publication [31].

Besides the traditional metal or bimetal overlay [47], some optical fiber SPR sensors use an additional high refractive index (RI) layer of metal oxides or other double-layer materials. It was proven that materials having high RI, such as TiO_2_, ZnO, ITO, InN, In_2_O_3_, and Ta_2_O_5_, improve the probe reliability and sensitivity and increase long-term stability [48].

For the purpose of the research, the tapered optical fibers probes and liquid crystal (LC) mixture 3092A (with RIs at T = 25 °C equal to *n_e_* = 1.5062; *n_o_* = 1.4507) were used as an external sensing medium [31]. Due to their birefringence, liquid crystals are used as an external cladding which allows the effective RI to be changed around the taper. As observed in the previous works, the initial mesogens arrangement has a significant influence on the resonant wavelength dip location [31]. In addition, a certain steering voltage range used to orient LC molecules U from 0 to 40 V allows changing the location of the resonant peak.

This paper presents the effect of a thin silver layer deposition on the tapered waist area. The choice of silver as an overlay was dictated by its high *ε_r_/ε_i_* ratio (Table 1), compared to the previously used Au material [31], therefore, an improvement in the results was expected [35,49]. A layer thickness of *d* = 10 nm was established during preliminary measurements. Furthermore, in the research, a comparison to the results obtained for gold layers [31] is included. The main parameters that were under consideration are resonant wavelength, peaks width, absorption, calibration plots, and SNR. In addition, the photos from the polarizing microscope are presented together with the corresponding oscilloscope images.

## 2. Materials and Methods

Tapered optical fibers were manufactured using the fiber-optic taper element technology) system. A detailed description of the FOTET and the manufacturing process is published in our previous works [24,50]. The obtained optical fiber tapers were characterized by the following parameters: length *l* = 25.0 ± 0.4 mm and attenuation α = 0.3 ± 0.1 dB @1550 nm for a single-mode fiber. Taper parameters were experimentally selected to obtain lower insertion losses, the possibility to build thick LCCs, and to reduce the applied LC steering voltage, as well as the highest sensitivity and influence of the Ag layer. Silver layers were deposited by using the EM SCD500 (Leica, Wetzlar, Germany) sputtering coater with the following parameters: chamber pressure *p* = 10^−2^ mbar and current *I* = 32 mA. Preliminary measurements showed that the thickness of silver layers (in contrast to the gold ones with thickness of *d* = 30 nm) had to be reduced. In subsequent studies, the thickness was established as *d* = 10 nm. The construction of the cell and the full specification of the materials used to manufacture the LCC is described in the following papers [24,31,50]. The obtained LCCs were filled with the LC mixture 3092A (Δ*n* = 0.056; Δ*ε* = 0.49). As mentioned earlier, the resonant wavelength peak depends on the LCC type, therefore, in this research, the three types of cells manufactured and taken under investigation were: orthogonal, parallel, and twist.

## 3. Results and Discussion

**Figure 3:**
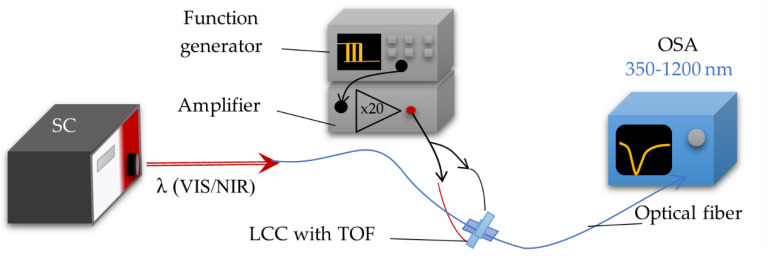
Measuring system setup used for spectral investigations.

To achieve the spectral characteristics, a measuring system setup was built and contained the supercontinuum—broadband light source SuperK EXTREME (NKT Photonics, Birkerød, Denmark) which operates on the wavelength range of VIS-NIR (400–2400 nm), optical spectrum analyzer (OSA) AQ6373B (Yokogawa, Tokyo, Japan) with a detection range of 350–1200 nm. The electric signal necessary for steering LCC was generated by the function generator DG1022Z (RIGOL, Beijing, China). The scheme of the measurement system is presented in Figure 3.

**Figure 4:**
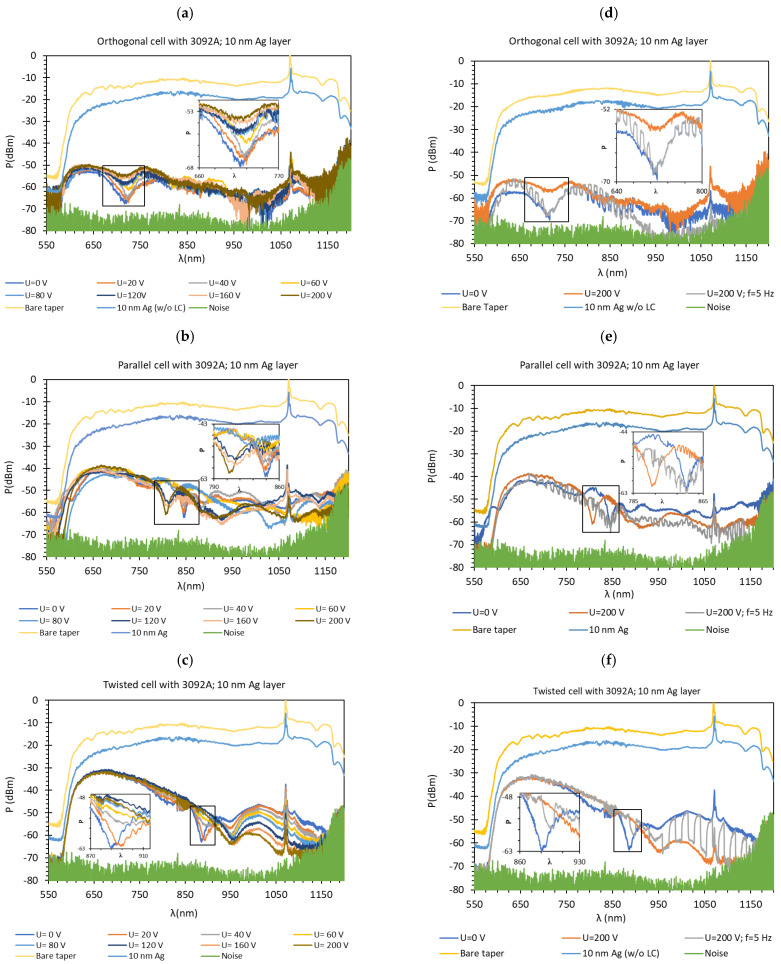
Optical power spectra obtained with 3092A-filled LCC with silver-covered TOF. Spectra in the left column were obtained for the voltage *U* from 0 to 200 V for LCC types: orthogonal (**a**), parallel (**b**), twist (**c**) without modulation. Spectra in the right column were obtained for cells under *U* = 0 and 200 V Hz for LCC types: orthogonal (**d**), parallel (**e**), twist (**f**) with signal modulation *f* = 5 Hz. Measurement provided at room temperature.

The measurements were carried out at room temperature with various steering voltage *U* from 0 to 200 V, without modulating the output signal and with amplitude modulation (AM) with a frequency of *f* equal to 5 Hz and 100% depth. The range of the steering voltage was the same for all LC types from 0 to 200 V. Based on the transmission spectra and images from the polarizing microscope, we can state that the threshold steering voltage is around 40 V for cells without a metallic layer and 20 V for cells with silver. It should be mentioned that the voltage needed to reorient molecules around the taper is much higher and is approximately equal to 180–200 V for which we obtained full reorientation of molecules. A wavelength *λ* range from 550 to 1200 nm was taken into account. Over this range, boundary conditions of TOF production and the additional cladding prevent waves propagation for all kinds of the presented cells. The spectral analysis was conducted for cells with three various molecules’ initial orientations. The results in Figure 4 present the spectral characteristics and the influence of the applied steering voltage *U* obtained for LC cells. In all graphs, the highest power (yellow color) corresponds to the spectra obtained for a bare optical fiber taper in the air, the blue color (below yellow) corresponds to spectra obtained for a tapered fiber covered with silver layer measure performed in the air, and the lowest power (green color) is the noise level of OSA. 

Firstly, as it can be observed, in contrast to gold, the silver layers cause a slightly higher decrease in the power transmitted in the tapered optical fiber (w/o LC) in relation to the given transmission in the bare taper. Moreover, in the case of the orthogonal and parallel cell, filling LCC causes higher attenuation in the whole wavelength range. For orthogonal cells, the maximum power is the lowest and does not exceed −50 dBm. For twisted cells, the obtained power is the highest and reaches its maximum at −30 dBm. An increase in attenuation for silver layers is probably caused by a higher extinction coefficient and the oxidation of silver [51]. The silver oxide formed by the oxidation process is characterized by different optical properties than the pure metal. Based on the literature [52], oxidation results in an increase in the absorption of visible light and a decrease in transmittance. It is well known that the oxidation phenomenon occurs as soon as silver is exposed to air and especially to water, which makes it difficult to yield a proper result. Moreover, during aging, the LC absorbs water due to a high dipole momentum. This results in a higher attenuation and deterioration of the probe [53].

In Figure 4a–c, for all cases, resonant dips occurred without the applied electric field. According to the expectations, for all cases, the resonant wavelengths dips shift in relation to the peaks obtained for tapers covered with gold layers. For the following types of cells: orthogonal, parallel, and twist, the resonant dips were established as 719 nm, 845 nm, and 886 nm, respectively. Increasing the steering voltage *U* in the range of 0–60 V (orthogonal cell) and 0–20 V (twisted cell) causes a slight red shift, however, further increase caused the disappearing of these peaks. In the case of the parallel cell, a blue shift is observed.

Figure 4d–f presents spectra obtained for *U* = 0 and 200 V with AM 100% depth with frequency *f* = 5 Hz, thus a dynamic response of cells can be observed. An interesting phenomenon is that in the case of the parallel and twisted cell, there is a lack of modulation for the short wavelengths range of 560–700 nm and 560–850 nm, respectively. This leads to the conclusion that for the shorter wavelengths for this type of cell, the difference between the RI of LC and TOF is too small, and it is harder to observe changes in the power during the switching ON/OFF. The operating wavelength range for all LCCs is similar to each other and, approximately, all cells operated in the whole measuring spectrum. The orthogonal cell, due to a higher attenuation for a longer wavelength, is barely at the detecting border.

**Figure 5:**
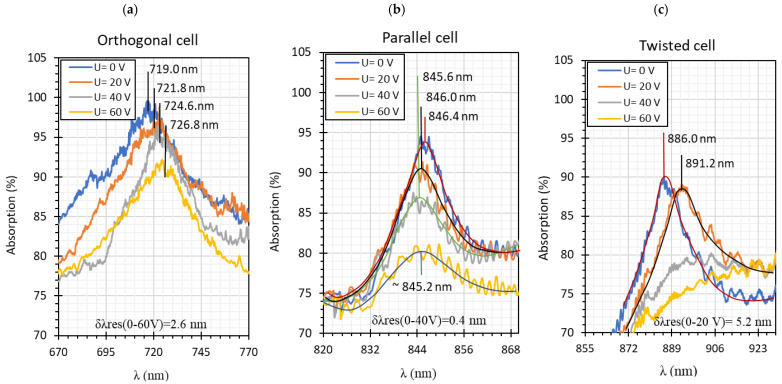
Resonant peaks shift obtained for: orthogonal (**a**), parallel (**b**), and twisted (**c**) cells.

The absorptions plots were also calculated and are presented in Figure 5a–c for orthogonal, parallel, and twisted cell types, respectively. According to the obtained results, the resonant dip of the orthogonal cell had the highest absorption (99%). In the case of other cells, the received absorption is higher than that obtained for cells with gold layers and equals 95% and 90%, respectively (for the gold layer—80%, 90%, and 70%, for orthogonal, parallel, and twist, respectively). Power oscillation is an important issue that makes it harder to measure the resonant peaks shifts (*δλ_res_*), however, it does not obscure the changes that are taking place which can be significantly observed in Figure 5. The deposited nano-metallic layer has high electric conductivity which causes an increase in charge density on the surface and oscillation of LC mesogens which results in a high fluctuation. To make possible the estimation of *δλ_res_*, the fitting cure was added, thus, obtained results should be taken as an approximation. Parameter *δλ_res_* in Figure 5 is an average value of the resonant peaks shift obtained between single voltage values: from 0 V to 20 V, from 20 V to 40 V, etc., and the values in the brackets correspond to steering voltage ranges which were considered to calculate *δλ_res_* for each LC cell type. Received *δλ_res_* for cells: orthogonal, parallel, and twist covered with silver layers were 2.6 nm, 0.4 nm, and 5.2 nm, respectively. In comparison to the same cells with gold layers, the *δλ_res_* increased about 1 nm ($\delta \lambda_{res}^{orthogonal}$ = 2.35 nm; $\delta \lambda_{res}^{twist}$ = 3.6 nm; $\delta \lambda_{res}^{parallel}$ = 0 nm). As one can see for parallel cells, the resonant wavelength decreases with the increase in voltage, which is different from the other two cases. In the authors opinion, blue shift implies that the effective RI around the taper slightly decreases which could be caused by the parallel (longer axis) arrangement of molecules and their higher anchoring to the surface.

The above results were used to estimate the differences in the obtained resonant dips. In Table 2 and Table 3, the following parameters are compared: type of LCC (orthogonal, parallel, twist), metal coverage (Au and Ag), *λ_res_*—resonant wavelength, Δ*λ*_0.5_—resonant dip width measured at 0.5 peak height, and SNR. In addition to, *δλ_res_*Ag-Au—resonant dip shift between peak Ag and Au, as well as *δλ*_0.5_—peak width difference between Ag and Au were calculated. It should be mentioned that due to the specific shape of the obtained spectra to the peak height calculations, lower power levels as a P_max_ of peaks (look at the Figure in Table 2 and Table 3) were taken into consideration. In the case of orthogonal and parallel cells, dips obtained without applied elective field (*U* = 0 V) are compared. It can be observed that for a parallel cell, two peaks are obtained (for *U* = 0 V and *U* = 200 V), thus; both were taken into consideration and additional parameters were estimated: Δ*λ_res_* (0–200 V)—the difference between peaks obtained for 0 and 200 V, and Δ*λ*_0.5_ (0–200 V)—the difference in width of peaks obtained for 0 and 200 V. The results contained in Table 2 and Table 3, corresponding to the LCC with gold, were taken or calculated based on a previous paper [31].

Comparing results from Table 2 and Table 3, it can be observed that the resonant peaks received for probes with Ag layers shifted approximately about 56.1 nm (calculated only for *U* = 0 V). In the case of the parallel cell, the additional parameter Δ*λ_res_* (0–200 V) shows that for both cases *λ_Ag_* (0–200 V) and *λ_Au_* (0–200 V), the difference between their peaks *λ* (0 V) and *λ* (200 V) is almost the same, and it is equal to approximately 37 nm. 

The next parameter under consideration is peak width. For both Au and Ag, the widest peak is obtained for the orthogonal cells. According to the literature [2,25], for silver covers, the width of resonant peaks measured at a height of 0.5 should be decreasing, which is observed. The maximum reduction has been received for a twisted cell, and it amounted to 67%. Interestingly, the calculated difference Δ*λ*_0.5_ (0–200 V) for a parallel cell shows that an increase in the electric field also causes an increase in the peak width. SNR is the last compared parameter and it was estimated by using data from Figure 5 and Table 2 and Table 3. For all LCC types with silver films, the SNR value was higher than for LCC with gold films, and the highest value was obtained for a twisted cell.

**Figure 6:**
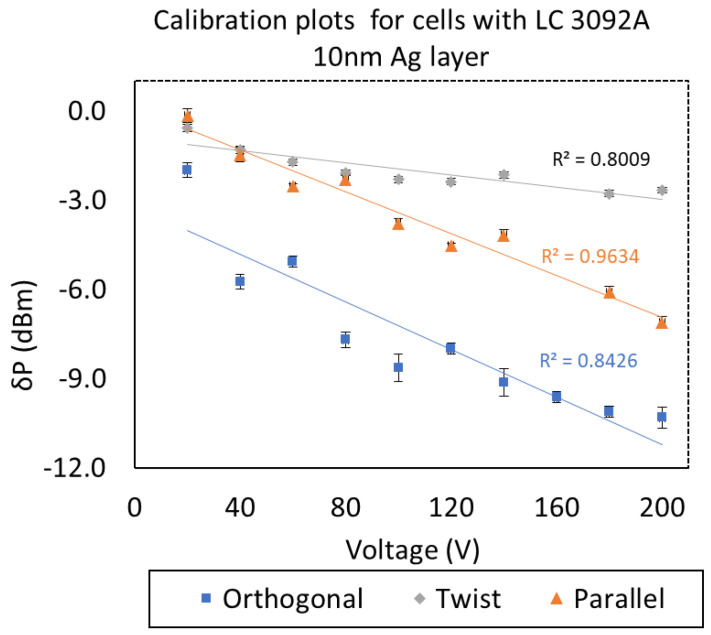
Calibration plots for cells with 3092A and the silver layer.

In the next step, calibration plots for cells with 3092A LC and taper covered by silver layer were made. Figure 6 presents the obtained results. Plots were calculated for the same wavelength value *λ* = 700 nm. In the case of LCCs with gold layers, the correlation value (R^2^) was equal to 0.82, 0.92, and 0.0013 for, orthogonal, twist, and parallel, respectively [31]. Comparing the obtained results in the previous work, it can be observed that R^2^ increased for orthogonal and parallel cells, however, the twisted cell slightly decreased.

**Figure 7:**
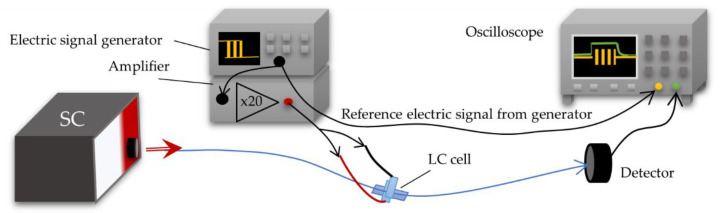
Dynamic response measuring system.

Table 4 contains the results of LCC dynamic characteristics and photos performed on the polarizing microscope (Olympus, Tokyo, Japan). The dynamic response measurements were performed in the measuring system containing an oscilloscope together with a detector as an optical analyzer (Figure 7).

The measurements were performed for LCC with/without deposited metal films, for steering voltage *U* from 0 to 200 V and AM with *f* = 1 Hz. The results are presented only for one LCC type. A switched-on state should be interpreted as a state in which the electric field between LCC electrodes is *E* > 0 V. Switching ON time is the time it takes the plot obtained for *U* = 200 V to reach 90% of its height from its minimum value and switching OFF time is the time it takes to reach 10% of its height from the maximum. Table 4 presents the dynamic characteristics of LCC with and without a metal layer and images from the polarizing microscope.

As observed in Table 4 (first column), the dynamic response changes between cells with/without a metal layer. In the case of LCC without a metallic layer, together with increasing the electric field, the measured voltage (LCC response from the detector) decreased. In contrast, the LCC with a metallic layer behaves quite differently and together with increasing the steering electric field, the measured voltage increases. This dependence is also visible at spectral measurements. The answer to this phenomenon can be observed in the images on the right in Table 4 (last column) (images for steering voltage *U* = 200 V). In the case of LCC without a deposited metal layer, an increase in the steering electric field for a certain range causes such a setting of the average LC director for which the RI value of LC is equal to the RI of the taper, causing reduced visibility of the tapered fiber in the LCC (see image in Table 4 for voltage *U* = 200 V). The metallic layer for deposition allows the increasing difference in RI between LC and TOF. As can be seen in the images obtained for cells with Ag and *U* = 200 V, the tapered fiber is continuously visible. Moreover, dynamic response, in the case of LCC in metallic, is characterized by high fluctuations. As was mentioned earlier, the deposited metallic layer causes increasing charge density on the surface. This phenomenon may be accompanied by ionization of LC and influences the ON/OFF switching times. The metallic layer significantly reduces the switching ON time, but on the other hand, ionization causes an increase in the relaxation time. Switching OFF time LCC with the metal layer is much longer than for LCC without a layer.

As is commonly known, various surfaces have a different impact on the LC molecules’ arrangement [54]. It is well established that mesogens nearest to the surface are anchored and can change the order of a few molecular layers. Analyzing the photos obtained without an applied electric field (*U* = 0 V), it can be observed that in both cases, around the tapered fiber n-director deformations (yellow circles) occur. This means that some areas around the fiber are characterized by a different arrangement of LC molecules than the molecules in the bulk. In this region, the effective refractive index (RI) is not identical to the other part of the cell. In the case of LCC without a metal layer, deformation is uniform (the same color on the whole length) in contrast to the taper covered with a metallic layer where deformation is heterogeneous. It can be assumed that the obtained glass surface of the tapered fiber (without covering) is uniform and clear, and mesogens are attached regularly by hydroxyl ions OH-. Silver is mechanically unstable, has low adhesion to glass, and has a natural tendency to form discontinuous islands. Moreover, depositing the metal layer by sputtering may be unregular or delaminate on the nanoscale, which is why inequalities are visible in the image. Deformation of n-director is highly undesirable because the evanescent field interacts with different effective RI in the tapered waist area. It should be mentioned that the extraordinary RI of 3092A is much higher than the fiber and, consequently, effective RI may be increased which may result in interrupting the SPR effect. In addition, black dots are visible on the upper images (in the red circles)—after-effects of the short circuit, however, if they are small and occur at a considerable distance from the tapered fiber their influence is negligible. Further images obtained for electric voltage *U* = 100 V show that surface-anchored mesogens are partially ordered and only their thin layer around is irregular. As was mentioned earlier, increasing the steering voltage up to 200 V causes ordering of the whole volume of LC molecules and vanishing deformation around the taper.

## 4. Conclusions

The performed research on the influence of a deposition thin silver layer on the tapered optical fiber surface and closed inside the LC cell allowed for the following conclusions to be drawn:

1. The resonant peaks shifted approximately about 55 nm in relation to the peaks obtained for gold layers. For the orthogonal and twisted cells, a red shift of the resonant dip was observed and they occurred at 720 nm and 887 nm, respectively. For the parallel cell, a blue shift was observed and the peak occurred at 845 nm.
2. The peaks width reduced to at least 50%, while SNR together with *δλ_res_* increased. The widest peak was obtained for the orthogonal cell.
3. The highest resonant peak absorption occurred for the orthogonal cell and equaled 99%. For the other two cases, absorption equaled a minimum of 90%.
4. Dynamic characteristics of LCC—with and without a metal layer—showed that the metallic layer caused a fluctuation, as well as an extension of the relaxation time.

This paper shows that most of the measured parameters of the constructed system have been improved. Consequently, the observed SPR effect was enhanced in relation to the previous work, however, it still requires further investigation to use these structures in real applications. It should be mentioned that during the measurements, some issues and additional effects occurred such as oxidation of the Ag layer, oscillation of LC mesogens, and signal overlapping. However, the obtained device has a high application potential, e.g., as a tunable filter for the chosen wavelengths. Furthermore, it could be used as a temperature, magnetic, and/or electric field sensor.

## Figures and Tables

**Table 1:** Permittivity parameters of typical metals used to overlay the SPR probes.

Metal	*ε_r_* + *iε_i_* *	*ε_r_/ε_i_* *	Probes Operating Wavelength Range	
Au	−11.36 + 1.23i	9.23	VIS	[35,36]
Ag	−18.22 + 0.48i	37.9	VIS	[37,38]
Cu	−12.46 + 0.65i	19.2	VIS-IR	[39,40]
Al	−43.42 + 15.40i	2.82	UV-NIR	[41,42]
Bi	−23.441 + 2.18i	10.7	UV-NIR	[43,44]
Pd	−15.27 + 15.17	1.01	NIR-IR	[45,46]

* Dielectric permittivity calculated for a 632.8 nm wavelength for all materials.

**Table 2:** Resonant dips comparison of the orthogonal and twisted cells.

Orthogonal Cell			Twisted Cell	
	Au	Ag	Au	Ag
*λ_res_*	*λ_res_* (0 V) = 665.8 nm	*λ_res_* (0 V) = 719.0 nm	*λ_res_* (0 V) = 831.4 nm	*λ_res_* (0 V) = 886.8 nm
*δλ_res_* Ag-Au	*δλ_res_* Ag-Au (0 V) = 53.2 nm		*δλ_res_* Ag-Au (0 V) = 55.4 nm	
Δ*λ*_0.5_	Δ*λ*_0.5_ (0 V) = 64.3 nm	Δ*λ*_0.5_ (0 V) = 33.2 nm	Δ*λ*_0.5_ (0 V) = 47.6 nm	Δ*λ*_0.5_ (0 V) = 15.4 nm
*δλ*_0.5_	*δλ*_0.5_ Ag-Au (0 V) = 33.1 nm → −51.5% *		*δλ*_0.5_ Ag-Au (0 V) = 32.2 nm → −67.6% *	
Resonant dip	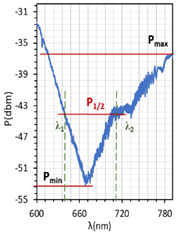	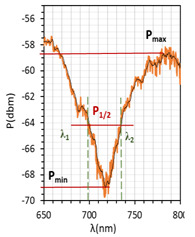	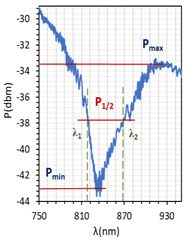	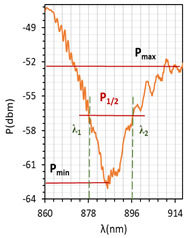
SNR	0.036	0.078	0.076	0.338

* Peaks width reduction in relation to Au layer.

**Table 3:** Resonant dips comparison of the parallel cell.

Parallel Cell				
	Au		Ag	
*λ_res_*	*λ_res_* (200 V) = 749.7 nm		*λ_res_* (200 V) = 807.2 nm	
	*λ_res_* (0 V) = 786.0 nm		*λ_res_* (0 V) = 845.6 nm	
	Δ*λ_res_* (0–200 V) = 36.3 nm		Δ*λ_res_* (0–200 V) = 38.4 nm	
*δλ_res_* Ag-Au	*δλ_res_* Ag-Au (200 V) = 57.5 nm			
	*δλ_res_* Ag-Au (0 V) = 59.6 nm			
Δ*λ*_0.5_	Δ*λ*_0.5_ (200 V) = 31.9 nm		Δ*λ*_0.5_ (200 V) = 16.2 nm	
	Δ*λ*_0.5_ (0 V) = 28.9 nm		Δ*λ*_0.5_ (0 V) = 12.2 nm	
	Δ*λ*_0.5_ (0–200 V) = 3 nm		Δ*λ*_0.5_ (0–200 V) = 4 nm	
*δλ*_0.5_	*δλ*_0.5_ Ag-Au (200 V) = 15.7 nm → −49.2% *			
	*δλ*_0.5_ Ag-Au (0 V) = 16.7 nm → −57.8% *			
Resonant dips	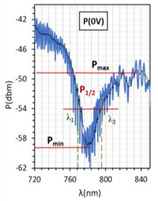	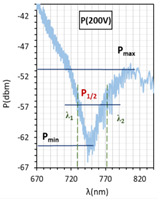	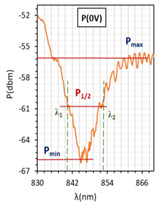	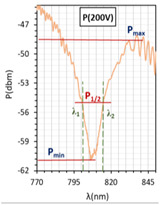
SNR	0.00 **		0.033	

* Peaks width reduction in relation to Au layer. ** *δλ_re_*_s_—this parameter could not be estimated.

**Table 4:** Dynamic characteristics of LCC and photos from the polarizing microscope.

	Dynamic Response of LC Cell	*U* = 0 V	*U* = 100 V	*U* = 200 V